# Alzheimer’s pathology is associated with altered cognition, brain volume, and plasma biomarker patterns in traumatic encephalopathy syndrome

**DOI:** 10.1186/s13195-023-01275-w

**Published:** 2023-07-21

**Authors:** Breton M. Asken, Jeremy A. Tanner, Leslie S. Gaynor, Lawren VandeVrede, William G. Mantyh, Kaitlin B. Casaletto, Adam M. Staffaroni, Corrina Fonseca, Ranjani Shankar, Harli Grant, Karen Smith, Argentina Lario Lago, Haiyan Xu, Renaud La Joie, Yann Cobigo, Howie Rosen, David C. Perry, Julio C. Rojas, Bruce L. Miller, Raquel C. Gardner, Kevin K. W. Wang, Joel H. Kramer, Gil D. Rabinovici

**Affiliations:** 1 Department of Clinical & Health Psychology, 1Florida Alzheimer’s Disease Research Center, University of Florida, 1225 Center Drive, Gainesville, FL 32610 USA; 2 Department of Neurology, Biggs Institute for Alzheimer’s and Neurodegenerative Diseases, South Texas Alzheimer’s Disease Research Center, University of Texas Health – San Antonio, 7703 Floyd Curl Drive, San Antonio, TX 78229 USA; 3 Department of Neurology, Weill Institute for Neurosciences, Memory and Aging Center, University of California, San Francisco, 675 Nelson Rising Lane, San Francisco, CA 94158 USA; 4 Department of Neurology, University of Minnesota, PWB 12-100, 516 Delaware Street SE, Minneapolis, MN 55455 USA; 5 Department of Neuroscience, Helen Wills Neuroscience Institute, University of California, 132 Barker Hall MC#3190, Berkeley, CA 94720 USA; 6 Department of Surgery, University of Florida, PO Box 100128, Gainesville, FL 32610 USA; 7 Sheba Medical Center, Tel Hashomer City of Health, Tel Aviv District, Derech Sheba 2, Ramat Gan, Israel; 8 Department of Neurobiology, Morehouse School of Medicine, 720 Westview Drive SW, Atlanta, GA 30310 USA

**Keywords:** Traumatic encephalopathy syndrome, Chronic traumatic encephalopathy, Amyloid, PET, Plasma, Biomarker, GFAP, NfL, Brain injury, Repetitive head impacts

## Abstract

**Background:**

Traumatic encephalopathy syndrome (TES) is a clinical phenotype sensitive but non-specific to underlying chronic traumatic encephalopathy (CTE) neuropathology. However, cognitive symptoms of TES overlap with Alzheimer’s disease (AD), and features of AD pathology like beta-amyloid (Aβ) plaques often co-occur with CTE, making clinical-to-pathological conclusions of TES diagnoses challenging. We investigated how Alzheimer’s neuropathological changes associated with cognition, brain volume, and plasma biomarkers in patients with repetitive head impacts (RHI)/TES, clinical AD, or typically aging controls.

**Methods:**

We studied 154 participants including 33 with RHI/TES (age 61.5 ± 11.5, 100% male, 11/33 Aβ[ +]), 62 with AD and no known prior RHI (age 67.1 ± 10.2, 48% male, 62/62 Aβ[ +]), and 59 healthy controls without RHI (HC; age 73.0 ± 6.2, 40% male, 0/59 Aβ[ +]). Patients completed neuropsychological testing (memory, executive functioning, language, visuospatial) and structural MRI (voxel-based morphometry analysis), and provided plasma samples analyzed for GFAP, NfL, IL-6, IFN-γ, and YKL-40. For cognition and plasma biomarkers, patients with RHI/TES were stratified as Aβ[ +] or Aβ[ −] and compared to each other plus the AD and HC groups (ANCOVA adjusting for age and sex). Differences with at least a medium effect size (Cohen’s *d* > 0.50) were interpreted as potentially meaningful.

**Results:**

Cognitively, within the TES group, Aβ[ +] RHI/TES performed worse than Aβ[-] RHI/TES on visuospatial (*p* = .04, *d* = 0.86) and memory testing (*p* = .07, *d* = 0.74). Comparing voxel-wise brain volume, both Aβ[ +] and Aβ[ −] RHI/TES had lower medial and anterior temporal lobe volume than HC and did not significantly differ from AD. Comparing plasma biomarkers, Aβ[ +] RHI/TES had higher plasma GFAP than HC (*p* = .01, *d* = 0.88) and did not significantly differ from AD. Conversely, Aβ[ −] RHI/TES had higher NfL than HC (*p* = .004, *d* = 0.93) and higher IL-6 than all other groups (*p*’s ≤ .004, *d*’s > 1.0).

**Conclusions:**

Presence of Alzheimer’s pathology in patients with RHI/TES is associated with altered cognitive and biomarker profiles. Patients with RHI/TES and positive Aβ-PET have cognitive and plasma biomarker changes that are more like patients with AD than patients with Aβ[ −] RHI/TES. Measuring well-validated Alzheimer’s biomarkers in patients with RHI/TES could improve interpretation of research findings and heighten precision in clinical management.

**Supplementary Information:**

The online version contains supplementary material available at 10.1186/s13195-023-01275-w.

## Introduction

Chronic traumatic encephalopathy (CTE) is a neurodegenerative tauopathy strongly associated with lifetime exposure to repetitive head impacts (RHI) [1, 2]. Traumatic encephalopathy syndrome (TES) is a proposed framework for identifying CTE pathology during life based on the degree of RHI and clinical symptoms [3]. Cognitive, neuroimaging, and other biomarker correlates of CTE neuropathology are not well defined. Lack of validated biomarkers for CTE and the high frequency of co-pathologies in brains with CTE complicate our ability to attribute clinical or biomarker changes observed during life in patients with RHI or TES to CTE pathology specifically.

Alzheimer’s disease (AD) is the most common neuropathological finding in patients with dementia. Older adults with prior RHI should have, at a minimum, similar age-related risk for AD as the general population with some data supporting increased risk or earlier age of AD symptom onset [4–6]. Neuritic beta-amyloid (Aβ) plaques, a hallmark neuropathological feature of AD, have been documented in over 70% of brains with widespread CTE [1] and are associated with older age at death [7]. AD is a critical differential diagnosis in patients with TES either as a primary cause of symptoms or as a contributor along with other diseases like CTE.

Memory loss and/or executive dysfunction are a core feature of TES commonly identified by informant reports of symptoms observed in patients later confirmed to have CTE at autopsy [1, 3, 8]. Amnestic and dysexecutive symptoms are also common manifestations of AD along with visuospatial dysfunction [9], which is less frequently associated with CTE [1]. Divergent cognitive profiles might be expected based on brain regions affected by AD versus CTE. CTE pathology predominantly affects frontal and temporal lobes with sparing of posterior regions until late in the course of severe cases [10, 11]. Emerging evidence shows frontotemporal atrophy patterns without clear posterior volume loss on MRI in patients with autopsy-confirmed CTE and without co-occurring AD [12, 13]. Conversely, posterior temporo-parietal regions are vulnerable to accumulating AD pathology [14]. Patients with RHI/TES harboring AD pathology (with or without CTE) may therefore have unique cognitive profiles and atrophy patterns compared to those without AD.

Fluid biomarkers measured in cerebrospinal fluid or blood are another way of characterizing the pathophysiology of neurodegenerative disease with several contexts of use including diagnosis, prognosis, clinical trial eligibility, and measuring treatment response. Greater cerebrospinal fluid levels of neurofilament light chain (NfL) and glial fibrillary acidic protein (GFAP) may relate to chronic symptoms after repeated concussions [15, 16]. Existing blood biomarker studies in older adults with prior extensive RHI have yielded inconsistent results [17, 18], which may be due in part to heterogeneous neuropathological processes across clinically-defined study cohorts (e.g., TES). Recent work in neurodegenerative disease populations shows that biomarkers like phosphorylated tau (e.g., P-tau181, P-tau217) [19] and glial fibrillary acidic protein (GFAP) are relatively specific to the Aβ-mediated components of AD pathophysiology [20–23] that presumably are distinct from CTE, including in patients with TES [19]. However, a wealth of data also support plasma GFAP as a marker of acute head trauma-related pathophysiology [24, 25]. Nonspecific neurodegenerative markers like plasma neurofilament light chain (NfL) show modest elevation in AD [22, 26, 27] and have unclear utility in patients with RHI/TES. Studies of patients with RHI/TES that do not account for AD pathology risk misattributing biomarker changes to CTE or other non-AD pathologies.

Implementing well-validated biomarkers for AD pathology into studies of patients with RHI/TES will advance our understanding and interpretation of symptom profiles and other biomarker findings. Phenotypic variability associated with AD in patients with RHI/TES has important implications for clinical management, expected benefits of disease-specific therapies, eligibility for clinical trials targeting AD, and prognostication. We compared neuropsychological testing, structural MRI, and a panel of plasma biomarkers between patients with RHI/TES, patients with AD, and healthy controls. Aβ PET status as a proxy for AD pathology and/or autopsy was completed for all participants, which allowed us to evaluate how presence of AD pathology in patients with TES impacted cognition, atrophy patterns, or plasma biomarkers both within TES and compared to AD or healthy controls.

## Methods

### Study participants

This study included research participants from the University of California, San Francisco (UCSF) Memory and Aging Center enrolled through either the UCSF Alzheimer’s Disease Research Center or healthy controls from the Brain Aging Network for Cognitive Health (BrANCH). Self-reported sociodemographic variables collected included age, sex, years of education, and race. Race categories (e.g., White, Black) were defined based on the US Office of Management and Budget’s Revisions to the standards for the Classification of Federal Data on Race and Ethnicity. Race reporting was consistent with the US National Institutes of Health policies. Race data were collected because adverse outcomes associated with social determinants of poor health may disproportionately impact underserved race groups [28, 29]. The predominance of White participants did not allow for appropriately examining differences based on race/ethnicity in this study.

Research participants with known RHI exposure through contact or collision sport participation or, if available, a neuropathological diagnosis of CTE, were considered for inclusion in the TES group. Participants underwent clinical evaluations including comprehensive history, neurologic exam, neuropsychological testing, caregiver interview and functional assessment (Clinical Dementia Rating scale; CDR), brain structural MRI, and blood draw. Consensus diagnoses were provided by a multidisciplinary team.

All identified participants with prior RHI were considered for the TES group and characterized retrospectively using the updated 2021 criteria [3]. Accordingly, we classified the likelihood of underlying CTE pathology in living individuals (“Suggestive of CTE,” “Possible CTE,” or “Probable CTE”) based on the degree of lifetime head trauma exposure, core clinical features, severity of functional impairment, and number of additional supportive features. Two investigators (BA and JT) independently classified each participant using revised TES criteria and then adjudicated discrepancies to reach consensus. TES participants in our cohort were all male and predominantly included former American football players. Given that all participants in this group had either known prior RHI or autopsy confirmation of CTE, but not all would necessarily meet research criteria for TES, we refer to this group as “RHI/TES” throughout.

AD participants met criteria for dementia [30] or MCI [9] due to AD, or presented with a non-memory predominant AD phenotype as described below. All AD participants had a positive Aβ-PET scan. A subset (*N* = 43) underwent tau PET with flortaucipir, of which 40 (93% of those with tau PET) had elevated signal in a metatemporal ROI based on a previously used quantitative threshold for AD-specific tau pathology (SUVR > 1.27) [31]. Since patients with TES often present with symptoms in their 60 s or earlier [3, 8], we included patients classified as early-onset AD (symptom onset before age 65; *N* = 36). Clinical phenotypes of patients in the AD group included single- or multi-domain amnestic (*N* = 45), posterior cortical atrophy [32] (*N* = 6), logopenic variant primary progressive aphasia [33] (*N* = 3), behavioral or dysexecutive [34, 35] (*N* = 2), corticobasal syndrome [36] (*N* = 1), and mixed/unspecified phenotype (*N* = 5). To minimize the likelihood of CTE co-pathology, we only included AD participants without a known history of prior traumatic brain injury or collision sport exposure. Prior head trauma exposure was determined through a review of medical history data collected through research on standardized forms including the National Alzheimer’s Coordinating Center Uniform Data Set Health History [37]. We additionally reviewed all medical history documented through neurologic examinations in clinical and research settings.

HC participants were clinically normal, functionally independent (CDR Global = 0), community-dwelling older adults participating in the UCSF BrANCH study. All participants were Aβ-PET negative and lacked cognitive symptoms or a history of neurologic, psychiatric, or other notable medical history like sleep apnea or stroke. Absence of repeated trauma and collision sport participation in the HC group was verified by completion of detailed self-report surveys of prior brain injury (Ohio State University TBI Identification Method [38]) and prior sport and military participation (Boston University Head Trauma Exposure Assessment [39]).

We used PET imaging and/or autopsy data (Additional file 1: Table 1) in this study to establish presence or absence of Aβ pathology for group classification. PET acquisition, processing, and interpretation details, as well as autopsy methods, are provided in Additional file 2.

### Neuropsychological testing

Cognitive test composite scores were created for episodic memory (California Verbal Learning Test short form, immediate and delayed recall; Benson figure recall), executive functioning (modified trail making test, lexical fluency, digit span backwards, Stroop inhibition, design fluency), language (animal fluency, 15-item Boston Naming Test), and visuospatial abilities (Benson figure copy, Number Location subtest of the Visual Object and Space Perception battery) [40]. Individual test raw scores were converted to z-scores based on the larger BrANCH study sample of clinically normal older adults (N per test = 231–763, 65 ± 13 years old, 60% female, 16.8 ± 2.4 years of education) and then averaged within each domain to create the composite score. For healthy controls in this study, only the Benson figure recall score was available for characterizing memory. Analyses comparing patients with TES to healthy controls were repeated using only the Benson figure recall score and results were consistent with those reported below.

### Voxel-wise structural neuroimaging

T1-weighted structural magnetic resonance imaging (MRI) scans were obtained on a 3.0 Tesla Siemens TIM Trio (35% of sample) or a 3.0 Tesla Siemens Prisma Fit (65% of sample) scanner. Magnetization-prepared rapid gradient-echo (MPRAGE) sequences were used to obtain whole brain T1-weighted images (1mm slice thickness). Whole brain voxel-based morphometry (VBM) analysis was performed using Statistical Parametric Mapping (SPM12, Wellcome Centre for Human Neuroimaging, London, UK). Pre-processing steps were completed using the SPM12 DARTEL (Diffeomorphic Anatomical Registration Through Exponentiated Lie Algebra) [41]. An average gray matter mask was created using only healthy control sample subjects. DARTEL was used to generate a sample-specific group template, then individual images were warped to that group template. Afterward, images in DARTEL space were normalized through linear registration to MNI (Montreal Neurological Institute) space and smoothed by an 8-mm full-width-at-half-maximum Gaussian kernel filter. More details are provided in Additional file 2.

### Plasma biomarkers

Venous blood was collected and stored in EDTA tubes (Alzheimer’s Disease Neuroimaging Initiative protocol) at − 80 °C at UCSF until being packed with dry ice and sent to the University of Florida for analysis following standard shipping protocols. We studied putative biomarkers for relevant pathophysiological processes including neuronal degeneration (NfL, total tau), astrocyte reactivity (GFAP), and inflammation (IL-6, YKL-40, IFN-γ). AD-specific plasma biomarkers (P-tau181, P-tau217) were reported previously [19]. Plasma GFAP, NfL, and total tau were measured via multiplex single molecule arrays on an SR-X analyzer (Simoa, Quanterix Neurology 4-Plex B). Plasma IL-6, YKL-40, and IFN-γ were measured with a chemiluminescence-based immunoassay using the Meso Scale Discovery platform (V-plex). All samples were analyzed in duplicate according to the manufacturer’s published protocols. We only included sample concentrations with coefficients of variance (CV) < 20% (excluded *N* = 1 GFAP, *N* = 1 NfL, *N* = 9 total tau, *N* = 3 IL-6, *N* = 2 YKL-40, *N* = 10 IFN-γ). Mean CV% for included plasma samples were 3.6% (GFAP), 3.6% (NfL), 6.8% (total tau), 5.6% (IL-6), 4.1% (YKL-40), and 6.0% (IFN-γ).

### Statistical analyses

Cognition and plasma biomarker analyses were performed using IBM SPSS version 28. We compared patients with RHI/TES, patients with AD, and healthy controls on cognitive test scores, brain volume, and plasma biomarker concentrations (log-transformed) using analyses of covariance (ANCOVA). All analyses included age and sex as covariates. Brain volume comparisons were additionally controlled for scanner and total intracranial volume. Cognitive test score comparisons also controlled for years of education. Planned pairwise post hoc comparisons were limited to RHI/TES vs. AD and TES vs. controls. To evaluate the role of Alzheimer’s disease pathology in patients with TES, analyses were then performed stratifying the RHI/TES group as Aβ[ +]and Aβ[ −]. A priori statistical significance was defined as *p* < 0.05. Group differences with at least a medium effect size (Cohen’s *d* > 0.50) [42] were interpreted as potentially meaningful given the small group sizes for some post hoc analyses and the likelihood of being underpowered to detect smaller effect sizes as statistically significant.

For VBM analyses, two-sample t-tests were performed to compare voxel-wise atrophy patterns between subgroups. Analyses included age, sex, scanner type, and TIV as covariates. A sample-specific explicit gray matter mask was applied to limit unnecessary voxel-wise comparison and increase sensitivity to true effects. Given the small sample size, uncorrected analyses with a statistical significance threshold of *p* < 0.001 and a cluster-level extent threshold of 30 is presented in the main manuscript. Additional analyses using a peak-level *p* < 0.05 after family-wise error (FWE) correction for multiple comparisons and cluster-level extent threshold of 0 is presented in Additional file 1.

## Results

### Participant characteristics

We retroactively applied recent TES research criteria to 33 participants with prior RHI. Diagnostic certainty level was *N* = 6 “Suggestive of CTE,” *N* = 9 “Possible CTE,” and *N* = 13 “Probable CTE.” We questioned whether TES criteria were met in 5 patients due to symptoms potentially being fully explained by another condition (*N* = 2 with behavioral variant frontotemporal dementia and motor neuron disease), RHI seemingly restricted to frequent falls later in life (*N* = 2), and no clear documentation of RHI details in existing clinical or research records (*N* = 1). A convenience sub-sample of *N* = 12 of the 33 participants with RHI/TES was analyzed at autopsy for neurodegenerative disease pathology, of which 9 had evidence of CTE neuropathology (*N* = 2 McKee Stage I, *N* = 5 Stage III, *N* = 2 Stage IV), including all 5 patients considered questionable for meeting new TES diagnostic criteria. Four out of 5 questionable TES cases were classified as Aβ[ −] RHI/TES. Study outcomes stratified by diagnostic certainty level are provided in Additional file 1: Figs. 1-3.

**Fig. 1:**
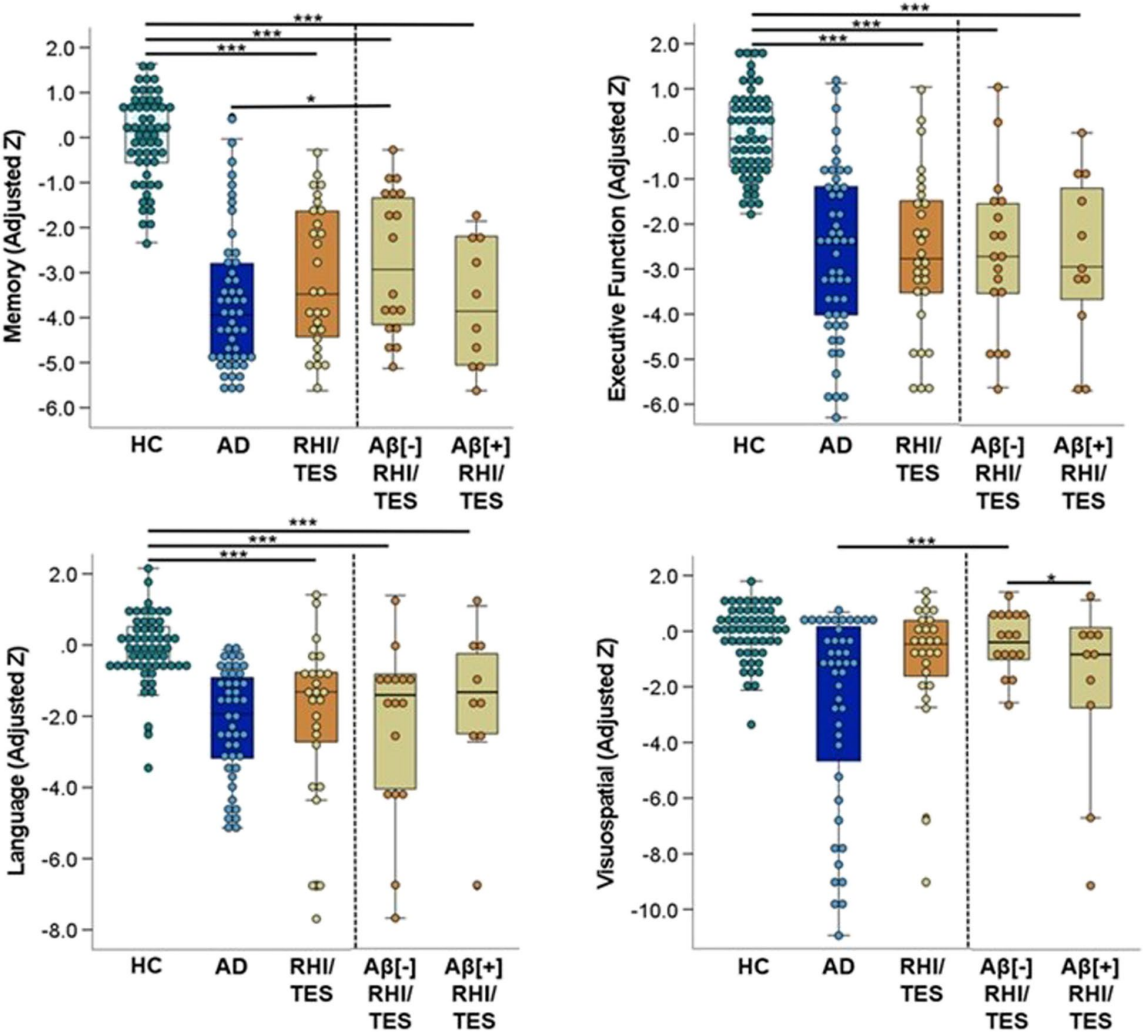
Group comparison of cognitive test scores by domain. Scores were adjusted for the effects of age, sex, and years of education observed in the healthy control (HC) group. Planned pairwise comparisons included patients with RHI/TES (with and without Aβ status stratification) vs. HC and vs. patients with Alzheimer’s disease (AD)

The total study sample was 154 participants (Table 1) including 33 patients with RHI/TES (100% male; 11/33 Aβ[ +]), 62 patients with AD (48% male; 62/62 Aβ[ +]), and 59 healthy controls (40% male; 0/59 Aβ[ +]). Patients with RHI/TES (age 61.5 ± 11.5 years) were significantly younger than patients with AD (age 67.1 ± 10.2; *p* = 0.005) and healthy controls (age 73.0 ± 6.2; *p* < 0.001). Patients with RHI/TES did not differ from patients with AD in clinical disease severity (global CDR; *p* = 0.90) but had significantly higher global cognitive score performance (Mini-Mental State Exam; 25.2 ± 4.7 vs. 22.9 ± 5.2, *p* = 0.009).

**Table 1:** Descriptive statistics of the study cohort stratified by patient group

	**Repetitive head impact/** **traumatic encephalopathy syndrome (RHI/TES)**			**Healthy controls**	**MCI/dementia due to AD**
	***Aβ( −)***	***Aβ(* +*)***	***All RHI/TES***		
***N***	22	11	33	59	62
**Age, years**	58.5 (12.1)	67.5 (7.6)	61.5 (11.5)	73.0 (6.2)	67.1 (10.2)
**Sex, *N* (%) Female**	0 (0)	0 (0)	0 (0)	35 (60)	32 (52)
**Education, years**	16.6 (1.8)	17.1 (1.4)	16.8 (1.7)	17.3 (2.3)	16.8 (2.6)
**Self-reported race, *N* (%)**					
* White*	18 (82)	10 (91)	28 (85)	53 (90)	52 (84)
* Black*	3 (14)	1 (9)	4 (12)	1 (2)	2 (3)
* Asian*	0 (0)	0 (0)	0 (0)	2 (3)	6 (10)
* Multiple*	1 (5)	0 (0)	1 (3)	3 (5)	2 (3)
**APOE genotype, *N* (%) e4**	2 (10)	3 (27)	5 (15)	10 (17)	32 (58)
**CDR-sum of boxes**	4.0 (3.1)	4.8 (2.6)	4.3 (2.9)	0.0 (0.1)	3.9 (2.3)
**CDR + FTLD sum of boxes**	5.3 (4.2)	6.1 (3.3)	5.6 (3.9)	0.0 (0.0)	4.4 (2.8)
**CDR-global**					
* 0*	3 (14)	0 (0)	3 (9)	59 (100)	0 (0)
* 0.5*	15 (68)	4 (36)	19 (58)	0 (0)	37 (60)
* 1.0* +	4 (18)	7 (64)	11 (33)	0 (0)	25 (40)
**Mini-Mental State Exam**	26.2 (2.5)	23.5 (7.0)	25.2 (4.7)	29.3 (0.7)	22.9 (5.2)
**American football, *N* (%)**	20 (91)	10 (91)	30 (91)	-	-
**Years of exposure**	12.4 (6.4)	15.4 (3.8)	13.4 (5.8)	-	-
**Aβ-PET, Centiloids**^**a**^	0.4 (16.7)	23.5 (24.3)	8.1 (22.0)	1.4 (10.3)	91.1 (34.6)
**Tau-PET (flortaucipir)**					
*** N***	12	6	18	-	43
** Metatemporal ROI SUVR**	1.16 (0.09)	1.56 (0.48)	1.31 (0.35)	-	2.01 (0.55)
** SUVR > 1.27, N(%)**	1	4	5	-	40

^a^Mean (SD) of Centiloids values reflects only scans performed within 2 years of other study outcomes (cognitive testing, structural imaging, blood draw)

Unadjusted cognitive test scores, brain volumes, and plasma biomarker concentrations are provided in Additional file 1: Table 2.

### Cognitive function

Patients with RHI/TES had significantly lower scores than healthy controls in memory (*p* < 0.001, *d* = 2.4), executive function (*p* < 0.001, *d* = 1.6), and language (*p* < 0.001, *d* = 1.2), but not visuospatial abilities (*p* = 0.54, *d* = 0.2). Compared to patients with AD, patients with RHI/TES had *better* scores on visuospatial abilities (*p* = 0.002, *d* = 0.85) and did not differ in other cognitive domains.

We then examined cognitive function in patients with RHI/TES separately based on amyloid status (Fig. 1). On visuospatial testing, Aβ[ +] RHI/TES performed worse than Aβ[ −] RHI/TES (*p* = 0.04, *d* = 0.86) and showed a trend towards lower scores than controls (*p* = 0.10, *d* = 0.62), but did not differ from patients with AD (*p* = 0.32, *d* = 0.36). Conversely, Aβ[ −] RHI/TES did not differ from controls on visuospatial testing (*p* = 0.49, *d* = 0.24) but performed better than patients with AD (*p* < 0.001, *d* = 1.2). There was also evidence that Aβ[ +] RHI/TES had lower memory scores than Aβ[ −] RHI/TES (*p* = 0.07, *d* = 0.74) and did not differ from patients with AD (*p* = 0.79, *d* = 0.1), while Aβ[ −] RHI/TES scored better on memory tests than patients with AD (*p* = 0.04, *d* = 0.64). Amyloid status of the patients with RHI/TES was not associated with other cognitive outcomes (Additional file 1: Table 3).

### Voxel-wise brain volume

Compared to healthy controls, patients with RHI/TES primarily exhibited lower brain volume in the anterior and medial temporal lobes and the thalamus. Less widespread volume loss was seen in the medial frontal and dorsal parietal regions. There were no statistically significant differences compared to AD. Using a more lenient statistical threshold (*p* < 0.01 uncorrected; 100 voxel threshold), patients with RHI/TES showed evidence of lower medial temporal, insula, and thalamus volumes than patients with AD.

**Fig. 2:**
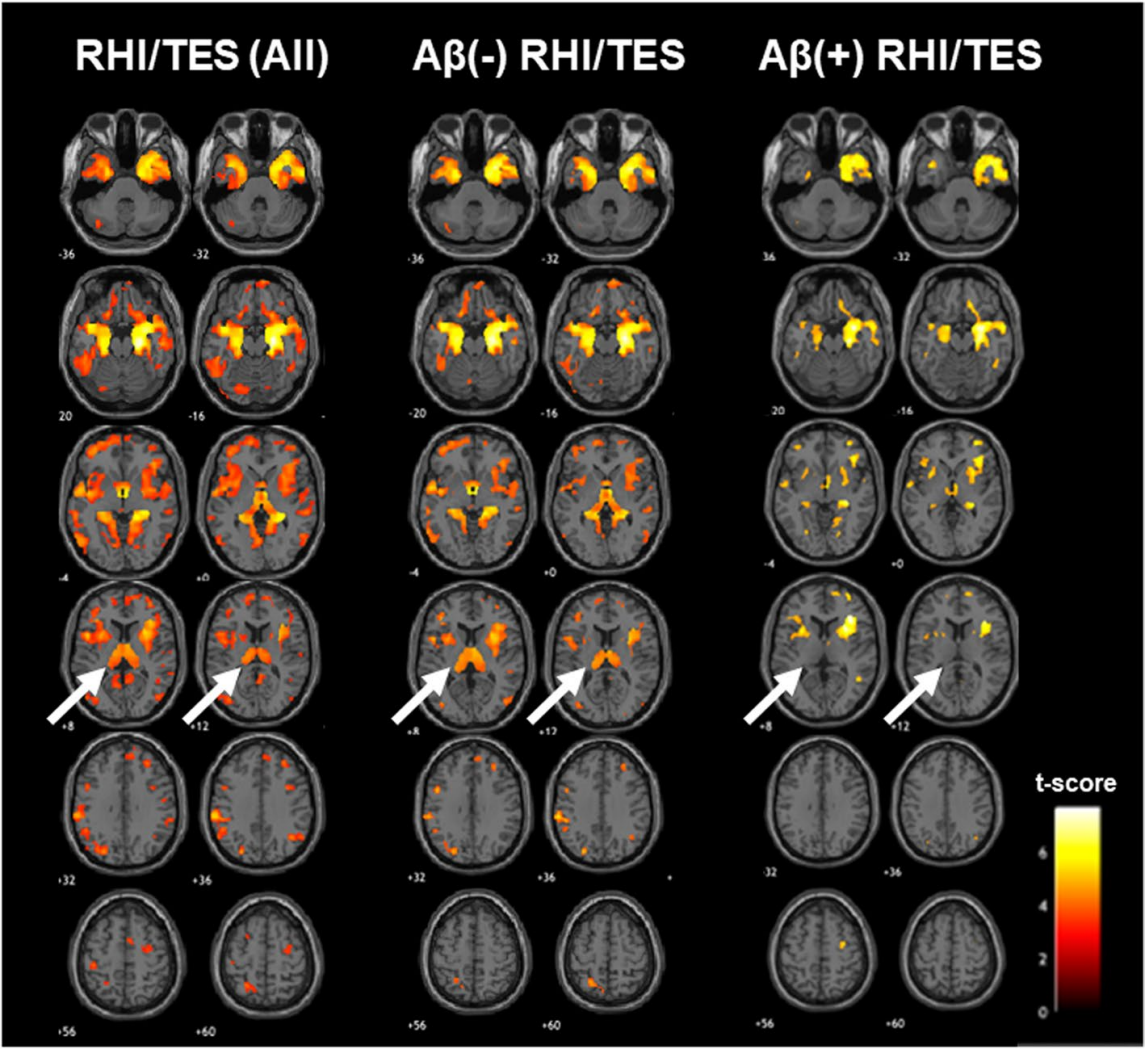
Voxel-based morphometry analyses comparing brain volume between repetitive head impact/traumatic encephalopathy syndrome (RHI/TES) study groups and healthy controls. Statistically significant volume differences (*p* < .001 uncorrected, voxel cluster minimum = 30) are represented by the red to yellow color spectrum, with the yellow end of the scale indicating greater (lower) volume difference from controls. Compared to controls, greatest and most consistent regional volume differences were in the anterior and medial temporal lobes regardless of Aβ status. Only Aβ( −) RHI/TES appeared to have lower thalamus volume than controls (white arrows). We did not observe statistically significant volume differences when comparing Aβ( +) to Aβ( −) RHI/TES or when comparing participants with RHI/TES to participants with AD

When stratifying patients with RHI/TES based on amyloid status (Fig. 2), both Aβ[ +] and Aβ[ −] RHI/TES primarily showed lower anterior and medial temporal volume than healthy controls, though only Aβ[ -] RHI/TES also had lower thalamus volume. Amyloid status of patients with RHI/TES was otherwise not clearly associated with different atrophy patterns. Additional multi-slice views and group comparisons with FWE correction are provided in Additional file 1: Figs. 4–6.

### Plasma biomarker concentrations

Compared to controls, patients with RHI/TES had significantly higher plasma GFAP (*p* = 0.03; *d* = 0.61), plasma NfL (*p* = 0.02, *d* = 0.67), and plasma IL-6 (*p* = 0.02, *d* = 0.67). Compared to patients with AD, patients with RHI/TES had lower plasma GFAP (*p* = 0.007, *d* = 0.68), higher plasma IL-6 (*p* = 0.007, *d* = 0.68), and did not differ in plasma NfL (*p* = 0.91, *d* < 0.10). Patients with RHI/TES did not have significantly different concentrations of plasma total tau, IFN-γ, or plasma YKL-40 than controls or patients with AD.

**Fig. 3:**
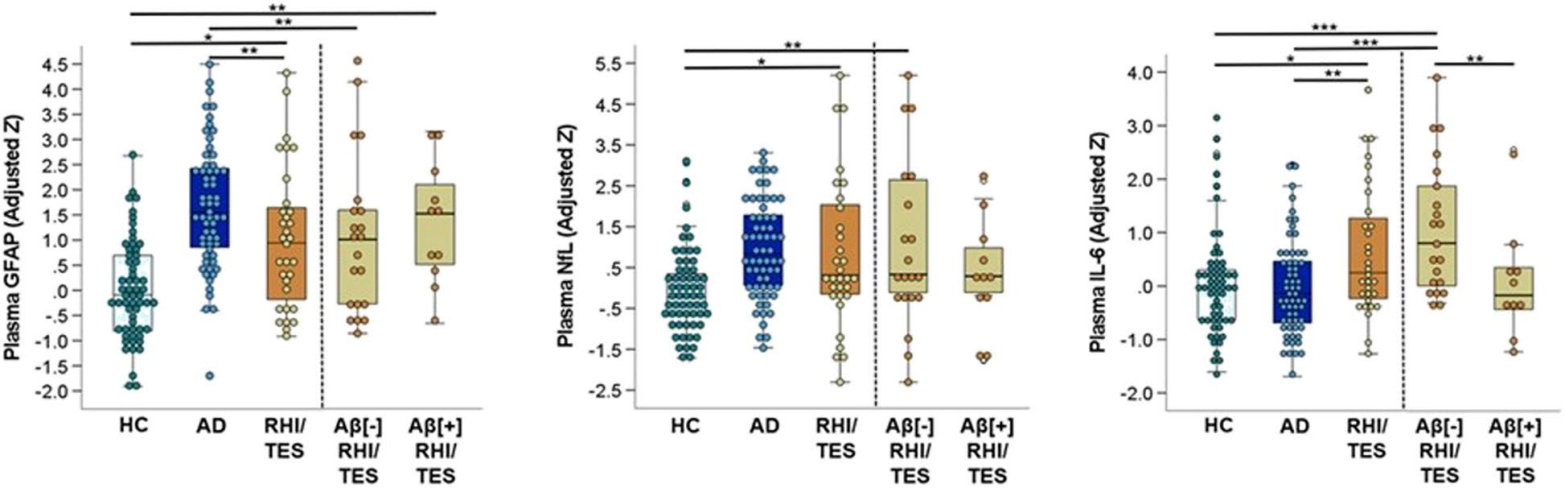
Group comparison of plasma biomarker concentrations. The dark black line within each box represents the median biomarker concentration and the upper and lower bounds of the box represent the interquartile range. Scores were adjusted for the effects of age and sex observed in the healthy control (HC) group. Planned pairwise comparisons included patients with RHI/TES (with and without Aβ status stratification) vs. HC and vs. patients with Alzheimer’s disease (AD). One patient with RHI/TES (Aβ[ −]) with very high NfL is not shown due to Y-axis distortion. Other plasma biomarkers analyzed include total tau, IFN-gamma, and YKL-40. No significant pairwise group differences were observed (data not shown in figure; see Supplemental Table 2)

When stratifying patients with RHI/TES based on amyloid status (Fig. 3), Aβ[ +] RHI/TES had higher plasma GFAP than controls (*p* = 0.01, *d* = 0.88) but did not differ from patients with AD (*p* = 0.26, *d* = 0.38), while Aβ[ −] RHI/TES did not differ from controls (*p* = 0.20, *d* = 0.40) and had lower plasma GFAP than patients with AD (*p* = 0.003, *d* = 0.86). Participant-level data showed a subset of Aβ[ −] RHI/TES had high plasma GFAP despite overall lower group concentrations. For plasma NfL, Aβ[ −] RHI/TES had significantly higher concentrations than controls (*p* = 0.004, d = 0.93) and a trend towards higher plasma NfL than Aβ[ +] RHI/TES (*p* = 0.11; *d* = 0.61). For IL-6, Aβ[ −] RHI/TES had higher concentrations than controls (*p* < 0.001, *d* = 1.1), Aβ[ +] RHI/TES (*p* = 0.004, *d* = 1.2), and patients with AD (*p* < 0.001, *d* = 1.1), while Aβ[ +] RHI/TES did not significantly differ from controls (*p* = 0.96, *d* < 0.10) or patients with AD (*p* = 0.91, *d* < 0.10).

We further explored how well plasma GFAP, NfL, and IL-6 differentiated study cohorts using area under the curve analysis and provide results in Additional file 1: Fig. 7. Classification accuracy largely reflected results for the group comparisons. Correlations between plasma biomarker concentrations and cognitive scores for the RHI/TES group are provided in Additional file 1: Table 4. The strongest correlations typically were observed between higher plasma GFAP and lower memory.

### Excluding atypical AD syndromes and questionable TES

Results were similar after excluding patients with AD presenting with predominantly non-amnestic clinical phenotypes (posterior cortical atrophy, logopenic variant primary progressive aphasia, behavioral, dysexecutive, corticobasal syndrome; *N* = 12). Results also did not change when excluding the 5 patients considered questionable for the most recent TES criteria.

## Discussion

In patients with RHI/TES, the patterns of cognitive difficulties, brain atrophy, and blood biomarker concentrations differ when there is evidence of Alzheimer’s pathology. Visuospatial dysfunction or elevated plasma GFAP may signal a subgroup of RHI/TES patients with Alzheimer’s disease pathology contributing to their symptoms (i.e., Aβ[ +] RHI/TES). Patients with RHI/TES who were Aβ[ −] did *not* differ from controls on visuospatial testing or plasma GFAP, but had higher plasma NfL than controls and higher plasma IL-6 than both controls and patients with AD. A temporal lobe-predominant pattern of brain volume loss was generally similar between Aβ[ +] and Aβ[ −] RHI/TES, though Aβ[ −] RHI/TES appeared to also exhibit thalamus atrophy. These findings provide preliminary support for understanding clinical heterogeneity of TES as possibly reflecting multiple proteinopathies.

Cognitive symptoms in patients with RHI/TES may point to affected brain regions and contributing neuropathology. The most common cognitive features of CTE reported retrospectively in a large autopsy series were memory loss and executive dysfunction [1]. Visuospatial dysfunction was the least commonly reported cognitive symptom [1], suggesting CTE pathology may not consistently disrupt brain regions supporting visuospatial function, such as the dorsal parietal lobe [12]. In contrast, the parietal lobe is among the most severely affected isocortical regions in patients with AD [14]. Patients with AD frequently develop visuospatial difficulties in the course of their disease with a subset reporting visuospatial problems as the first and most prominent symptom (i.e., posterior cortical atrophy due to AD [32]). Our data show that patients with RHI/TES and objective visuospatial deficits may be more likely to have AD contributing to their disease course. We did not observe clear parietal atrophy in the Aβ[ +] RHI/TES group compared to controls except for a few isolated voxel clusters. This may reflect the Aβ[ +] RHI/TES group being evaluated early in the disease course. Compared to participants with AD, the Aβ[ +] RHI/TBI group had a much lower cortical Aβ burden (mean CLs = 24 vs. 91) and the subset of Aβ[ +] RHI/TBI participants with tau PET also had a lower disease burden than the group with AD (mean metatemporal SUVR = 1.56 vs. 2.01).

There was notable within-group variability in plasma biomarker concentrations, but divergent patterns emerged that further support the relevance of AD pathology in patients with RHI/TES. Plasma GFAP, a putative marker of astrocyte reactivity to disease [43], was higher in patients with RHI/TES overall than controls, but this was driven by Aβ[ +] RHI/TES patients. Plasma GFAP is tightly linked to AD-related Aβ plaques [21, 23, 44] with less consistent evidence of elevations in non-AD dementia cohorts [45, 46]. However, beyond these group-level findings, we inspected individual participant data, and a subset of Aβ[ −] RHI/TES patients had relatively high plasma GFAP concentrations. More work is needed to determine whether the history of RHI, CTE, or other non-AD pathologies in patients with TES is associated with higher plasma GFAP concentrations.

We also observed group differences in plasma NfL and IL-6 that were driven by the Aβ[ −] RHI/TES group. We cannot determine with our study design whether higher levels in Aβ[ −] RHI/TES than healthy controls (NfL and IL-6) and patients with AD (IL-6) reflects CTE pathology. Additional possibilities for higher NfL in Aβ[ −] RHI/TES include the presence of other dysregulated proteins like TDP-43. TDP-43 proteinopathy, often limbic-predominant, is also linked with repetitive head trauma [1, 13, 47–49] and is associated with severe neurodegeneration such as hippocampal sclerosis [50, 51]. This aligns with our finding of Aβ[ −] RHI/TES patients having lower hippocampal volume than controls and not differing significantly from Aβ[ +] RHI/TES or AD, though medial temporal structures are also impacted by CTE [11]. Limbic-predominant TDP-43 is also commonly comorbid with AD [52]. Regarding IL-6 elevation in Aβ[ −] RHI/TES, systemic inflammation is chronically dysregulated in a subset of patients with repetitive head trauma [53, 54] and may lead to a neurologic impairment through non-AD pathways, but this requires much further investigation. Ultimately, improving detection of multiple non-AD pathologies during life (CTE, TDP-43 proteinopathy, inflammation, etc.) will substantially advance our understanding of biomarker correlates in patients with RHI/TES.

The goal of the recently proposed TES diagnostic framework is to aid in identifying CTE pathology during life [3]. TES criteria require a history of “substantial” head trauma exposure plus objective deficits on memory or executive function testing, with or without neurobehavioral symptoms. Additional symptoms such as visuospatial dysfunction do not preclude a diagnosis unless it is felt that another condition “fully accounts” for observed symptoms. Reaching this conclusion is challenging because of the high rates of co-pathologies in patients with cognitive impairment or dementia and because neuroimaging or fluid biomarker data do not factor into the current TES criteria. It is reasonable to expect that many patients without CTE, or with CTE plus comorbid pathologies, will meet the currently proposed criteria for TES, including patients with AD who have sufficient prior head trauma exposure. Whether patients with TES and evidence of AD pathology represent a clinically or neuropathologically distinct subgroup of AD remains an open and important question with direct implications for prognosis, patient management, and disease-specific clinical trials.

Key strengths of our study included comprehensive neuropsychological testing, neuroimaging, and plasma biomarker collection in a well-characterized group of patients with RHI/TES with biomarker or neuropathologic confirmation of Alzheimer’s pathology. While meeting diagnostic criteria for TES implies enrichment for CTE pathology, we cannot confirm or determine the degree to which CTE pathology contributed to observed group differences in this study. Clinico-pathological studies that can link clinical and biomarker data during life with neuropathological findings postmortem are required. In the meantime, measuring well-validated AD biomarkers in patients with RHI/TES may help explain heterogeneous cognitive and biomarker outcomes and inform whether TES “subtypes” reflecting different combinations of symptoms or underlying neuropathology is useful.

Our study was limited by the relatively small sample of patients and the cross-sectional design. These findings are considered preliminary given that we likely were underpowered to detect potentially meaningful group differences as statistically significant, especially for voxel-wise brain volume comparisons. Our sample overall had minimal racial diversity and the observed relationships may not generalize to traditionally underrepresented race groups at higher risk for suffering the negative effects of social determinants of poor health (e.g., structural and systematic racism). This is particularly relevant for professional American football players who are considered at the highest risk for TES or CTE and disproportionately self-identify as Black compared to the general population. Our RHI/TES group was all male and there is a tremendous need to better understand the association of repetitive head trauma and later-life cognitive outcomes among females. For the AD group, we relied on medical records and less sensitive medical history questionnaires from research visits to rule out head trauma. This approach may underestimate actual lifetime exposure, especially youth or adolescent collision sport experiences that are unlikely to be documented or ruled out systematically. Individual cognitive domains differed in the number of included tests and performance on some tests is influenced by difficulties in other domains (e.g., animal fluency influenced by both language and executive function). While the inclusion of early-onset AD patients was beneficial for comparison to similarly-aged patients with TES, early-onset AD patients may have different cognitive profiles and disease pathophysiology than late-onset AD. We attempted to address this limitation through additional analyses that excluded patients with atypical manifestations of AD, which did not meaningfully change the results. Lastly, most patients in the AD group had biomarker confirmation of both significant Aβ and tau burden, and quantification of pathology burden based on PET suggested that the AD group had more severe AD pathology than the Aβ[ +] patients with RHI/TES. Direct comparisons of the Aβ[ +] RHI/TES and AD groups are interpreted cautiously given the likely imbalance of underlying disease severity.

## Conclusions

Presence of Alzheimer’s pathology in patients with RHI/TES is associated with altered cognitive and biomarker profiles. Measuring well-validated Alzheimer’s biomarkers in patients with RHI/TES could improve interpretation of research findings and heighten precision in clinical management. Larger clinico-pathological studies are needed to determine the impact of Alzheimer’s and other neuropathologies on symptom and biomarker trajectories in patients with RHI/TES, with or without CTE.

## Supplementary Information

**Additional file 1:** **Supplemental Table 1.** Autopsy-based designation of Aβ pathology for a subset of study participants without Aβ-PET available. **Supplemental Table 2.** Unadjusted raw values for cognitive, brain volume, and plasma biomarker concentrations for each study group. Data are presented as both mean (standard deviation) and median (interquartile range). **Supplemental Table 3.** Pairwise group comparison effect sizes (Cohen’s d) for cognition and plasma biomarker concentrations. Bolded effect sizes were statistically significant (*p*<.05). For comparisons between groups of patients with RHI/TES and healthy controls (HC) and patients with Alzheimer’s disease (AD), the direction of the effect size (positive or negative effect size) is relative to the groups of patients with RHI/TES (i.e., negative effect size = lower value for RHI/TES group, positive effect size = higher value for RHI/TES group). For within RHI/TES group comparisons (Aβ[+] vs. Aβ[-]), direction of the effect size is relative to the Aβ[+] RHI/TES group (i.e., negative effect size = lower value for Aβ[+] RHI/TES, positive effect size = higher value for Aβ[+] RHI/TES). Suggested effect size magnitude interpretations (Cohen, 1994): d>[0.8] (Large), [0.5]<d<[0.8] (Medium), [0.2]<d<[0.5] (Small), d<[0.2] (negligible). **Supplemental Table 4.** Pearson’s correlations between plasma concentrations of GFAP, NfL, and IL-6 with cognitive test scores. Values represent Pearson’s r correlation strength. Plasma biomarkers were age- and sex-adjusted and cognitive composite scores were age-, sex-, and education-adjusted based on demographic associations observed in our healthy controls. **Supplemental Figure 1.** Cognitive test scores stratified by diagnostic certainty for chronic traumatic encephalopathy (CTE) according to 2021 research criteria (Katz et al., 2021). Test scores were adjusted for effects of age, sex, and years of education observed in the healthy control group. “Questionable TES” refers to participants with symptoms potentially being fully explained by another condition, repetitive head impacts seemingly restricted to frequent falls later in life, or no clear documentation of RHI in existing clinical or research records (all with autopsy-confirmed CTE). **Supplemental Figure 2.** Region of interest (ROI) brain volumes stratified by diagnostic certainty for chronic traumatic encephalopathy (CTE) according to 2021 research criteria (Katz et al., 2021). Brain volumes were adjusted for effects of age, sex, total intracranial volume, and scanner observed in the healthy control group. “Questionable TES” refers to participants with symptoms potentially being fully explained by another condition, repetitive head impacts seemingly restricted to frequent falls later in life, or no clear documentation of RHI in existing clinical or research records (all with autopsy-confirmed CTE). **Supplemental Figure 3.** Plasma biomarker concentrations stratified by diagnostic certainty for chronic traumatic encephalopathy (CTE) according to 2021 research criteria (Katz et al., 2021). Biomarker concentrations were adjusted for effects of age and sex observed in the healthy control group. “Questionable TES” refers to participants with symptoms potentially being fully explained by another condition, repetitive head impacts seemingly restricted to frequent falls later in life, or no clear documentation of RHI in existing clinical or research records (all with autopsy-confirmed CTE). One patient with TES (“Questionable”) with very high NfL is not shown due to Y-axis distortion. Data for plasma total tau, IFN-gamma, or plasma YKL-40 did not differ between any study groups and are not shown. **Supplemental Figure 4.** Multi-slice view of voxel-based morphometry analysis comparing the overall RHI/TES cohort to healthy controls. The left panel shows voxel-wise volume differences based on *p*<.001 uncorrected threshold (minimum voxels=30) and the right shows voxels that remained significant (*p*<.05) with family-wise error correction applied. Uncorrected thresholds were interpreted given the relatively small sample sizes. **Supplemental Figure 5.** Multi-slice view of voxel-based morphometry analysis comparing the Aβ(-) RHI/TES cohort to healthy controls. The left panel shows voxel-wise volume differences based on *p*<.001 uncorrected threshold (minimum voxels=30) and the right shows voxels that remained significant (*p*<.05) with family-wise error correction applied. Uncorrected thresholds were interpreted given the relatively small sample sizes.** Supplemental Figure 6.** Multi-slice view of voxel-based morphometry analysis comparing the Aβ(+) RHI/TES cohort to healthy controls. The left panel shows voxel-wise volume differences based on *p*<.001 uncorrected threshold (minimum voxels=30) and the right shows voxels that remained significant (*p*<.05) with family-wise error correction applied. Uncorrected thresholds were interpreted given the relatively small sample sizes. **Supplemental Figure 7.** Area under the curve (AUC) analysis showing differentiation of RHI/TES group(s) from healthy control (HC) and Alzheimer’s disease (AD) cohorts. Age- and sex-adjusted plasma biomarker levels were calculated based on demographic effects observed in the HC group. For all comparison, the RHI/TES group is the positive state (i.e., AUC > 0.5 reflects higher concentrations associated with RHI/TES classification). For the within RHI/TES comparison, the Aβ(+) RHI/TES group is the positive state. The table shows the AUC values with 95% confidence intervals for all pairwise comparisons.

**Additional file 2.** 

## Data Availability

All study data are available on reasonable request made to the UCSF Memory and Aging Center. Academic, not-for-profit investigators can request data for professional education and for research studies. Requests can be made online (https://memory.ucsf.edu/research-trials/professional/open-science). Datasets used for the analyses for the current study are also available from the corresponding author on reasonable request.

## Abbreviations

Aβ: Beta-amyloid
AD: Alzheimer’s disease
CDR: Clinical Dementia Rating scale
CTE: Chronic traumatic encephalopathy
GFAP: Glial fibrillary acidic protein
HC: Healthy controls
IFN-γ: Interferon gamma
IL-6: Interleukin 6
NfL: Neurofilament light chain
PET: Positron emission tomography
RHI: Repetitive head impacts
TDP43: Transactive response DNA binding protein of 43 kDa
TES: Traumatic encephalopathy syndrome
