# Supplementary material for: Alzheimer’s pathology is associated with altered cognition, brain volume, and plasma biomarker patterns in traumatic encephalopathy syndrome
Source: Alzheimers Res Ther. 2023 Jul 21;15:126. doi: 10.1186/s13195-023-01275-w (PMC10360257; doi:10.1186/s13195-023-01275-w)
Supplement: Supplementary file 1 — Additional file 1: Supplemental Table 1. Autopsy-based designation of Aβ pathology for a subset of study participants without Aβ-PET available. Supplemental Table 2. Unadjusted raw values for cognitive, brain volume, and plasma biomarker concentrations for each study group. Data are presented as both mean (standard deviation) and median (interquartile range). Supplemental Table 3. Pairwise group comparison effect sizes (Cohen’s d) for cognition and plasma biomarker concentrations. Bolded effect sizes were statistically significant (p<.05). For comparisons between groups of patients with RHI/TES and healthy controls (HC) and patients with Alzheimer’s disease (AD), the direction of the effect size (positive or negative effect size) is relative to the groups of patients with RHI/TES (i.e., negative effect size = lower value for RHI/TES group, positive effect size = higher value for RHI/TES group). For within RHI/TES group comparisons (Aβ[+] vs. Aβ[-]), direction of the effect size is relative to the Aβ[+] RHI/TES group (i.e., negative effect size = lower value for Aβ[+] RHI/TES, positive effect size = higher value for Aβ[+] RHI/TES). Suggested effect size magnitude interpretations (Cohen, 1994): d>[0.8] (Large), [0.5]<d<[0.8] (Medium), [0.2]<d<[0.5] (Small), d<[0.2] (negligible). Supplemental Table 4. Pearson’s correlations between plasma concentrations of GFAP, NfL, and IL-6 with cognitive test scores. Values represent Pearson’s r correlation strength. Plasma biomarkers were age- and sex-adjusted and cognitive composite scores were age-, sex-, and education-adjusted based on demographic associations observed in our healthy controls. Supplemental Figure 1. Cognitive test scores stratified by diagnostic certainty for chronic traumatic encephalopathy (CTE) according to 2021 research criteria (Katz et al., 2021). Test scores were adjusted for effects of age, sex, and years of education observed in the healthy control group. “Questionable TES” refers to participants with s [file 13195_2023_1275_MOESM1_ESM.pdf]

Study Cohort	Time from Evaluation to Death (years)	Thal Phase (Aβ)	CTE Stage
Aβ(+) RHI/TES	<1	3	I
Aβ(+) RHI/TES	7	5	III
Aβ(-) RHI/TES	<1	0	III
Aβ(-) RHI/TES	2	0	IV
Aβ(-) RHI/TES	5	1	0*
Aβ(-) RHI/TES	2	1	III
Aβ(-) RHI/TES	4	1	III
AD	1	4	0
AD	<1	4	0
AD	2	5	0

*Neuropathological findings of apparent focal traumatic tauopathy restricted to astroglia and not fulfilling 2021 NINDS criteria for CTE neuropathology

Supplemental Table 1. Autopsy-based designation of Aβ pathology for a subset of study participants without Aβ-PET available.

	Repetitive Head Impact/ Traumatic Encephalopathy Syndrome (RHI/TES)			Healthy Controls	MCI/Dementia due to AD
	Aβ(-)	***Aβ(+)***	***All RHI/TES***		
**Cognitive Testing (z), N**	20	11	31	57	60
Memory	-1.7 (1.6)<br>-1.4 (-3.2 – -0.2)	-2.8 (1.4)<br>-2.8 (-4.3 – -1.5)	-2.0 (1.6)<br>-1.9 (-3.4 – -0.5)	0.2 (1.0)<br>0.2 (-0.2 – 0.8)	-3.0 (1.4)<br>-3.2 (-4.2 – -2.4)
Executive Function	-1.0 (1.1)<br>-1.1 (-1.7 – -0.4)	-1.2 (1.2)<br>-1.4 (-2.0 – -0.2)	-1.1 (1.1)<br>-1.2 (-1.8 – -0.4)	0.3 (0.7)<br>0.3 (-0.2 – 0.7)	-1.4 (1.1)<br>-1.5 (-2.3 – -0.4)
Language	-1.5 (2.1)<br>-0.8 (-2.9 – -0.3)	-1.1 (2.1)<br>-0.3 (-1.9 – 0.1)	-1.4 (2.1)<br>-0.7 (-1.9 – -0.1)	0.2 (0.7)<br>0.2 (-0.1 – 0.6)	-1.6 (1.6)<br>-1.2 (-2.6 – -0.2)
Visuospatial	-0.1 (0.7)<br>0.2 (-0.6 – 0.6)	-1.5 (2.5)<br>-0.4 (-2.6 – 0.1)	-0.6 (1.7)<br>-0.2 (-0.7 – 0.2)	0.0 (0.7)<br>0.1 (-0.3 – 0.6)	-2.1 (2.8)<br>-0.9 (-3.9 – 0.1)
**Grey Matter Volume, mL**	18	8	26	44	42
Frontal	125.1 (18.9)<br>125.8 (114.9-136.3)	120.6 (23.1)<br>125.4 (110.4-134.2)	123.7 (19.9)<br>125.8 (114.9-134.7)	125.4 (12.5)<br>122.8 (117.2-134.6)	120.0 (18.1)<br>118.4 (106.3-131.9)
Temporal	98.9 (15.3)<br>97.7 (89.3-109.5)	95.6 (21.9)<br>97.9 (85.4-111.8)	97.9 (17.2)<br>97.7 (87.7-109.5)	99.8 (10.8)<br>98.8 (91.1-105.7)	91.0 (13.9)<br>91.5 (79.7-101.7)
Parietal	89.8 (11.0)<br>91.5 (81.0-98.1)	84.7 (16.3)<br>87.3 (76.3-98.9)	88.3 (12.7)<br>90.5 (80.1-98.1)	88.2 (8.0)<br>87.6 (82.1-95.0)	81.3 (13.0)<br>81.0 (72.6-89.7)
Occipital	42.0 (6.0)<br>42.2 (38.2-45.9)	40.5 (6.9)<br>41.7 (36.3-44.9)	41.5 (6.2)<br>42.2 (38.0-45.3)	39.3 (4.5)<br>38.3 (36.1-43.0)	36.4 (6.4)<br>36.2 (31.5-41.5)
Hippocampus	4.7 (0.9)<br>4.6 (4.3-5.5)	4.6 (1.1)<br>4.5 (3.6-5.8)	4.7 (0.9)<br>4.6 (3.9-5.6)	5.2 (0.5)<br>5.2 (4.8-5.6)	4.6 (0.7)<br>4.6 (4.2-5.1)
Subcortical	25.5 (3.2)<br>26.0 (23.1-27.7)	24.6 (2.5)<br>23.5 (23.3-27.0)	25.2 (3.0)<br>24.9 (23.2-27.6)	25.4 (2.6)<br>24.9 (23.5-26.8)	24.6 (2.6)<br>24.1 (22.7-26.3)
**Plasma Biomarkers (pg/mL), N**	21	11	32	59	62
GFAP	169 (126)<br>110 (73-245)	245 (174)<br>183 (135-296)	195 (146)<br>144 (85-265)	199 (113)<br>179 (123-243)	335 (185)<br>285 (212-420)
NfL	25.5 (39.0)<br>12.0 (7.5-26.1)	15.1 (9.2)<br>10.9 (9.7-19.6)	21.9 (32.2)<br>11.9 (8.5-19.7)	14.0 (6.3)<br>12.8 (10.3-15.5)	17.7 (8.3)<br>15.3 (11.7-21.6)
Total Tau	1.3 (0.6)<br>1.2 (0.7-1.7)	1.4 (0.7)<br>1.4 (0.7-2.2)	1.3 (0.6)<br>1.3 (0.7-1.7)	1.5 (0.9)<br>1.3 (1.0-2.0)	1.7 (0.8)<br>1.6 (1.2-2.1)
IL-6	1.1 (1.1)<br>0.72 (0.40-1.53)	0.6 (0.6)<br>0.45 (0.28-0.65)	0.9 (1.0)<br>0.52 (0.36-1.16)	0.7 (0.7)<br>0.49 (0.34-0.61)	0.6 (0.5)<br>0.43 (0.28-0.66)
YKL-40 ($x10^3$)	16.1 (8.8)<br>13.3 (10.5-18.0)	22.2 (11.1)<br>19.9 (11.8-31.6)	18.1 (9.9)<br>14.9 (11.1-21.3)	21.4 (13.9)<br>17.9 (14.0-22.8)	22.5 (16.5)<br>16.6 (12.4-26.6)

IFN-gamma	3.0 (5.0)<br>1.9 (1.3-2.6)	2.5 (1.7)<br>2.3 (1.2-4.2)	2.8 (4.1)<br>1.9 (1.3-2.7)	3.9 (4.3)<br>2.8 (1.7-4.6)	2.5 (2.3)<br>1.8 (1.2-3.1)

Supplemental Table 2: Unadjusted raw values for cognitive, brain volume, and plasma biomarker concentrations for each study group. Data are presented as both mean (standard deviation) and median (interquartile range).

	Cognition							
	**Memory**		**Executive Function**		**Language**		**Visuospatial**	
	HC	*AD*	*HC*	*AD*	*HC*	*AD*	*HC*	*AD*
**All RHI/TES**	**-2.3**	0.4	**-1.5**	0.1	**-1.3**	-0.1	-0.1	**1.0**
**Aβ[-] RHI/TES**	**-2.0**	**0.8**	**-1.5**	0.2	**-1.5**	-0.3	0.2	**1.2**
**Aβ[+] RHI/TES**	**-2.8**	-0.1	**-1.6**	-0.1	**-1.0**	0.2	-0.6	0.4
**Aβ[+] vs. Aβ[-]**	-0.7		-0.1		0.4		**-0.9**	

	Plasma Biomarker Concentrations											
	**GFAP**		**NfL**		**Total Tau**		**IL-6**		**IFN-gamma**		**YKL-40**	
	HC	*AD*	*HC*	*AD*	*HC*	*AD*	*HC*	*AD*	*HC*	*AD*	*HC*	*AD*
**All RHI/TES**	**0.6**	**-0.7**	**0.7**	<[0.1]	-0.1	-0.4	**0.7**	**0.7**	-0.1	0.3	0.1	-0.1
**Aβ[-] RHI/TES**	0.4	**-0.9**	**0.9**	0.3	-0.2	-0.5	**1.1**	**1.1**	-0.1	0.4	-0.1	-0.2
**Aβ[+] RHI/TES**	**0.9**	-0.4	0.3	-0.4	<[0.1]	-0.3	<[0.1]	<[0.1]	-0.2	0.3	0.3	0.1
**Aβ[+] vs. Aβ[-]**	0.5		-0.6		0.2		**-1.2**		0.1		0.3	

Supplemental Table 3: Pairwise group comparison effect sizes (Cohen's d) for cognition and plasma biomarker concentrations. Bolded effect sizes were statistically significant (p<.05). For comparisons between groups of patients with RHI/TES and healthy controls (HC) and patients with Alzheimer's disease (AD), the direction of the effect size (positive or negative effect size) is relative to the groups of patients with RHI/TES (i.e., negative effect size = lower value for RHI/TES group, positive effect size = higher value for RHI/TES group). For within RHI/TES group comparisons (Aβ[+] vs. Aβ[-]), direction of the effect size is relative to the Aβ[+] RHI/TES group (i.e., negative effect size = lower value for Aβ[+] RHI/TES, positive effect size = higher value for Aβ[+] RHI/TES). Suggested effect size magnitude interpretations (Cohen, 1994): d≥[0.8] (Large), [0.5]≤d<[0.8] (Medium), [0.2]≤d<[0.5] (Small), d<[0.2] (negligible)

	Memory			Executive Function			Language			Visuospatial		
	All	***Aβ(+)***	***Aβ(-)***	***All***	***Aβ(+)***	***Aβ(-)***	***All***	***Aβ(+)***	***Aβ(-)***	***All***	***Aβ(+)***	***Aβ(-)***
**GFAP**	-.51	-.47	-.58	-.11	-.02	-.23	-.27	.07	-.48	-.29	-.35	-.11
**NfL**	-.24	-.10	-.39	-.04	-.11	-.02	-.39	.19	-.58	-.09	-.33	-.13
**IL-6**	.08	.29	-.17	-.02	-.25	.21	-.10	-.15	-.03	-.05	-.41	-.24

Supplemental Table 4: Pearson's correlations between plasma concentrations of GFAP, NfL, and IL-6 with cognitive test scores. Values represent Pearson's r correlation strength. Plasma biomarkers were age- and sex-adjusted and cognitive composite scores were age-, sex-, and education-adjusted based on demographic associations observed in our healthy controls.

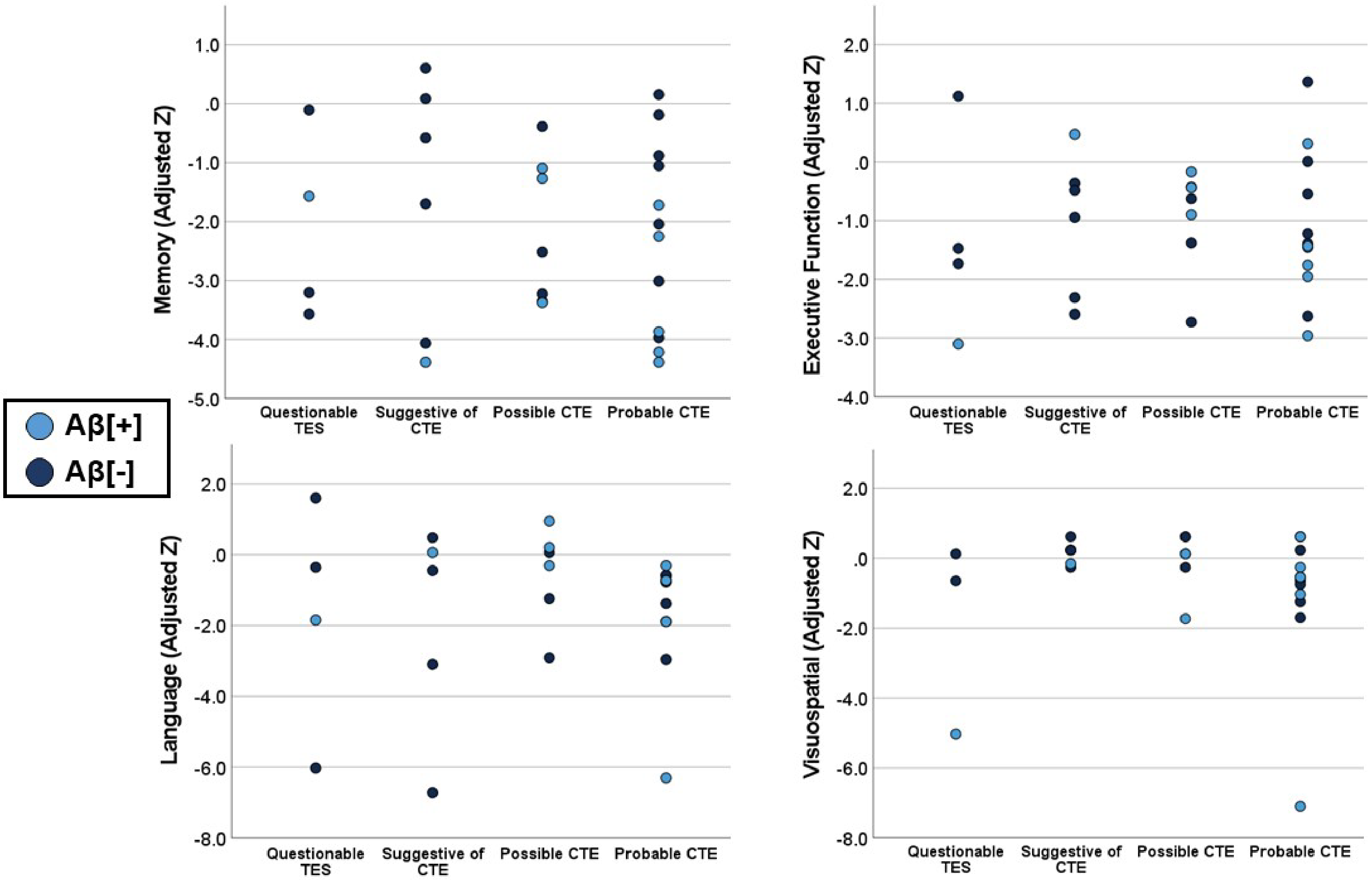

Supplemental Figure 1: Cognitive test scores stratified by diagnostic certainty for chronic traumatic encephalopathy (CTE) according to 2021 research criteria (Katz et al., 2021). Test scores were adjusted for effects of age, sex, and years of education observed in the healthy control group. "Questionable TES" refers to participants with symptoms potentially being fully explained by another condition, repetitive head impacts seemingly restricted to frequent falls later in life, or no clear documentation of RHI in existing clinical or research records (all with autopsy-confirmed CTE).

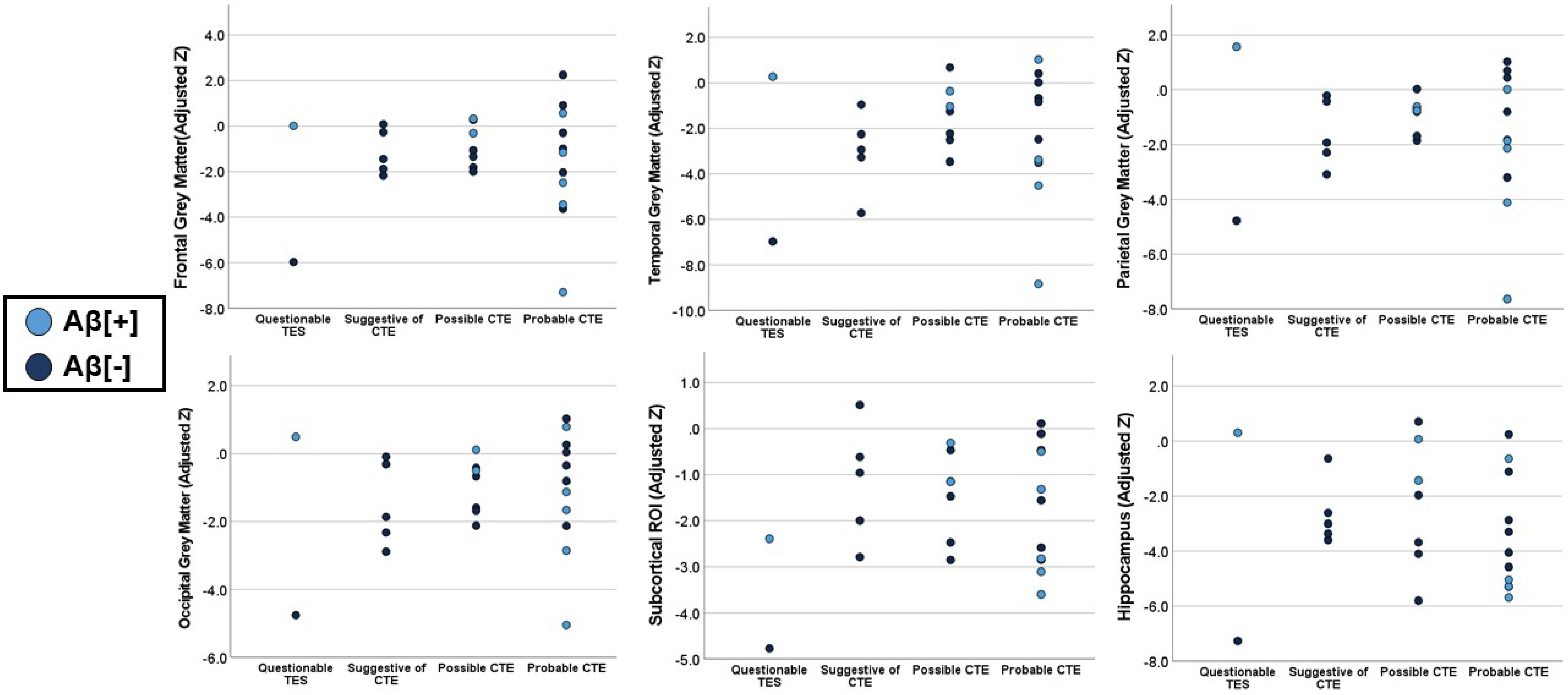

Supplemental Figure 2: Region of interest (ROI) brain volumes stratified by diagnostic certainty for chronic traumatic encephalopathy (CTE) according to 2021 research criteria (Katz et al., 2021). Brain volumes were adjusted for effects of age, sex, total intracranial volume, and scanner observed in the healthy control group. "Questionable TES" refers to participants with symptoms potentially being fully explained by another condition, repetitive head impacts seemingly restricted to frequent falls later in life, or no clear documentation of RHI in existing clinical or research records (all with autopsy-confirmed CTE).

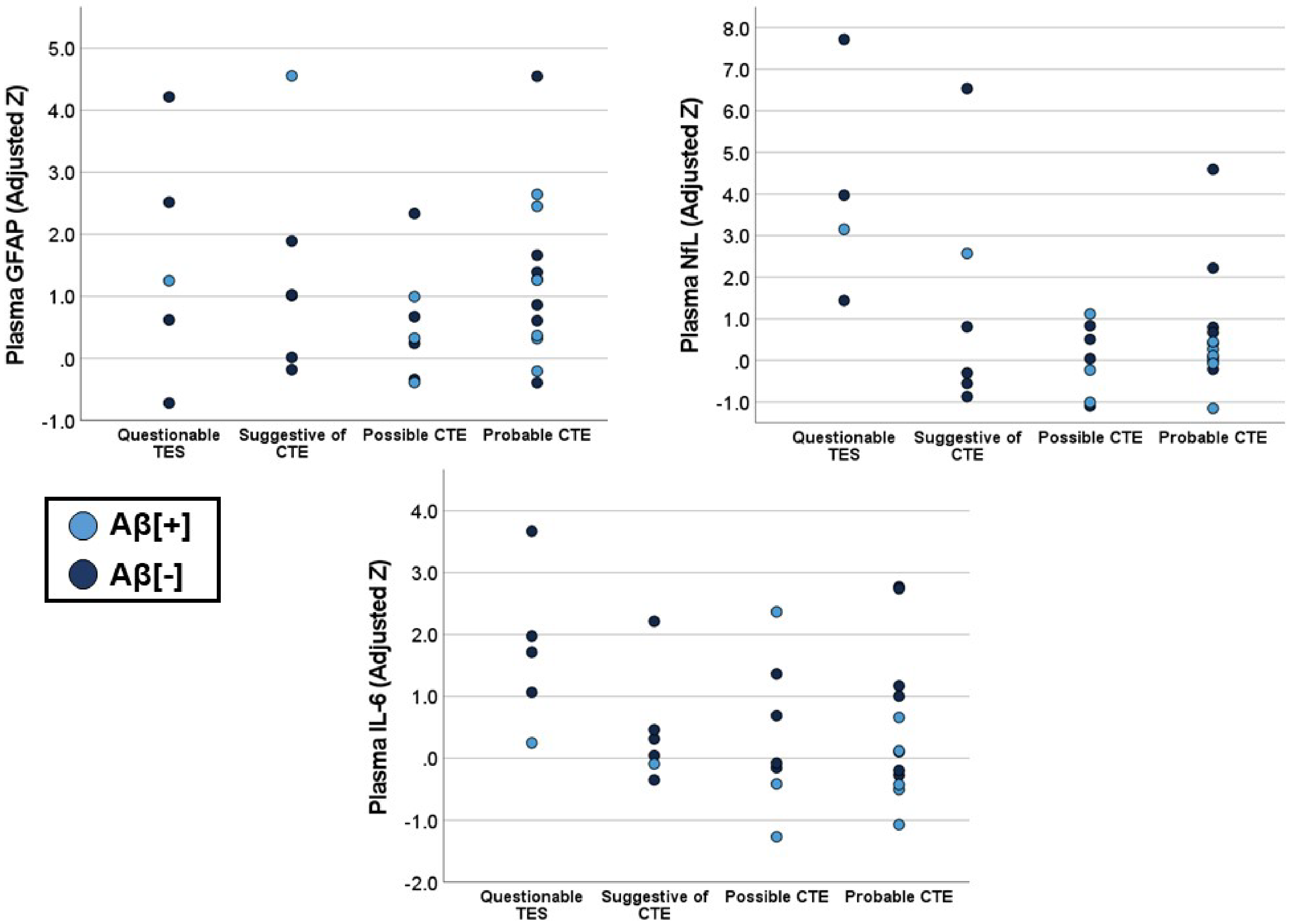

Supplemental Figure 3: Plasma biomarker concentrations stratified by diagnostic certainty for chronic traumatic encephalopathy (CTE) according to 2021 research criteria (Katz et al., 2021). Biomarker concentrations were adjusted for effects of age and sex observed in the healthy control group. "Questionable TES" refers to participants with symptoms potentially being fully explained by another condition, repetitive head impacts seemingly restricted to frequent falls later in life, or no clear documentation of RHI in existing clinical or research records (all with autopsy-confirmed CTE). One patient with TES ("Questionable") with very high NfL is not shown due to Y-axis distortion. Data for plasma total tau, IFN-gamma, or plasma YKL-40 did not differ between any study groups and are not shown.

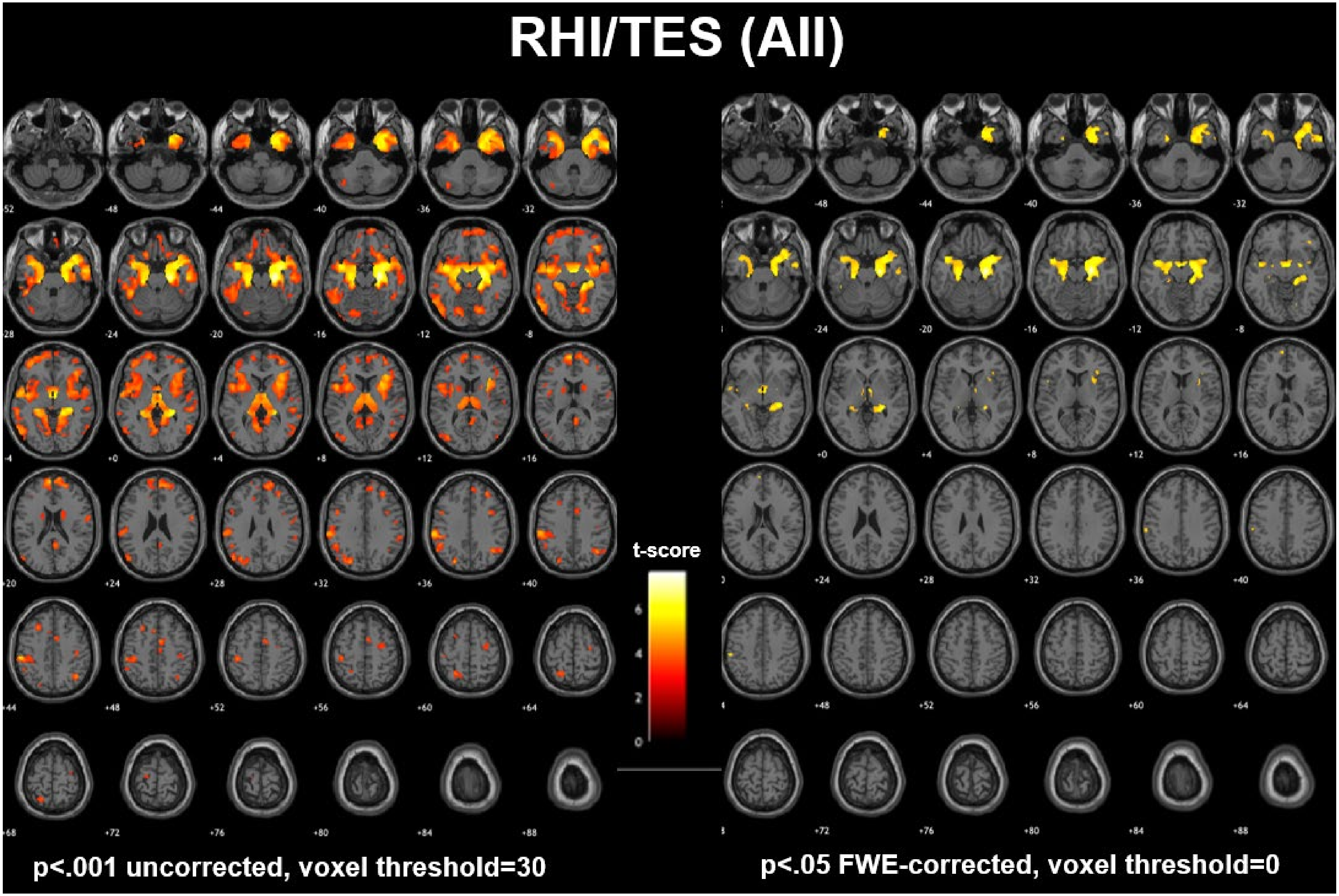

Supplemental Figure 4: Multi-slice view of voxel-based morphometry analysis comparing the overall RHI/TES cohort to healthy controls. The left panel shows voxel-wise volume differences based on p<.001 uncorrected threshold (minimum voxels=30) and the right shows voxels that remained significant (p<.05) with family-wise error correction applied. Uncorrected thresholds were interpreted given the relatively small sample sizes.

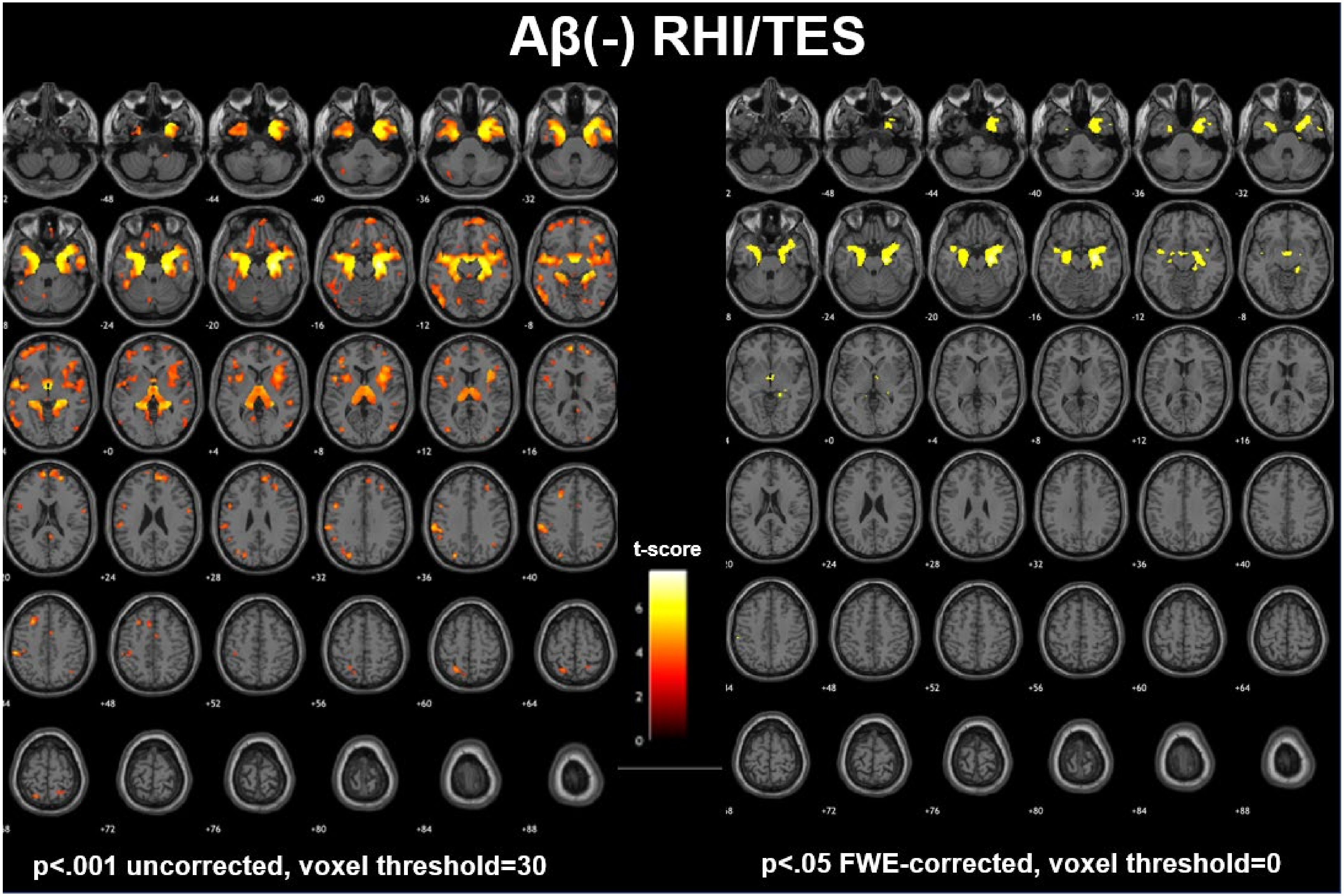

Supplemental Figure 5: Multi-slice view of voxel-based morphometry analysis comparing the Aβ(-) RHI/TES cohort to healthy controls. The left panel shows voxel-wise volume differences based on $p<.001$ uncorrected threshold (minimum voxels=30) and the right shows voxels that remained significant ($p<.05$) with family-wise error correction applied. Uncorrected thresholds were interpreted given the relatively small sample sizes.

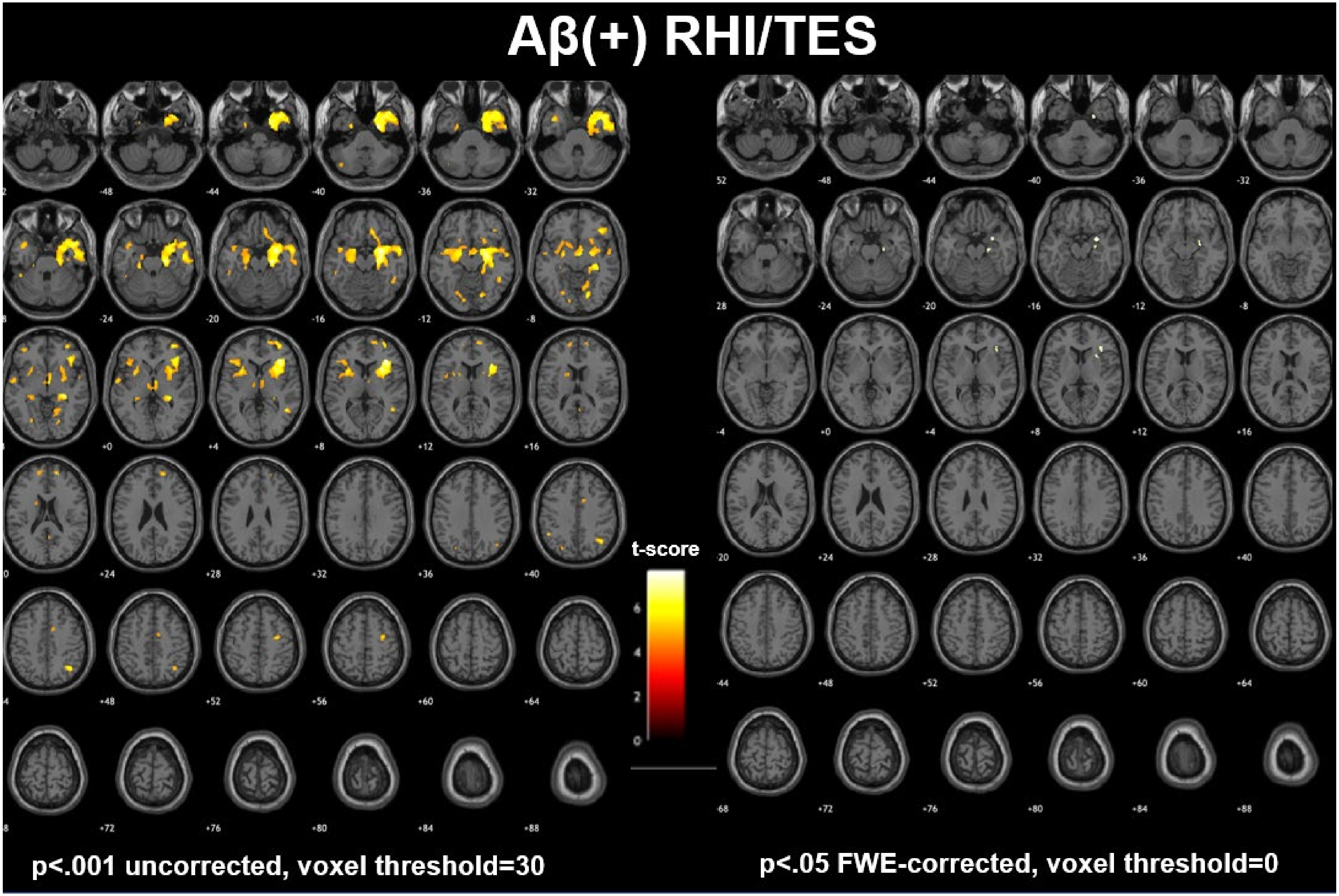

Supplemental Figure 6: Multi-slice view of voxel-based morphometry analysis comparing the Aβ(+) RHI/TES cohort to healthy controls. The left panel shows voxel-wise volume differences based on p<.001 uncorrected threshold (minimum voxels=30) and the right shows voxels that remained significant (p<.05) with family-wise error correction applied. Uncorrected thresholds were interpreted given the relatively small sample sizes.

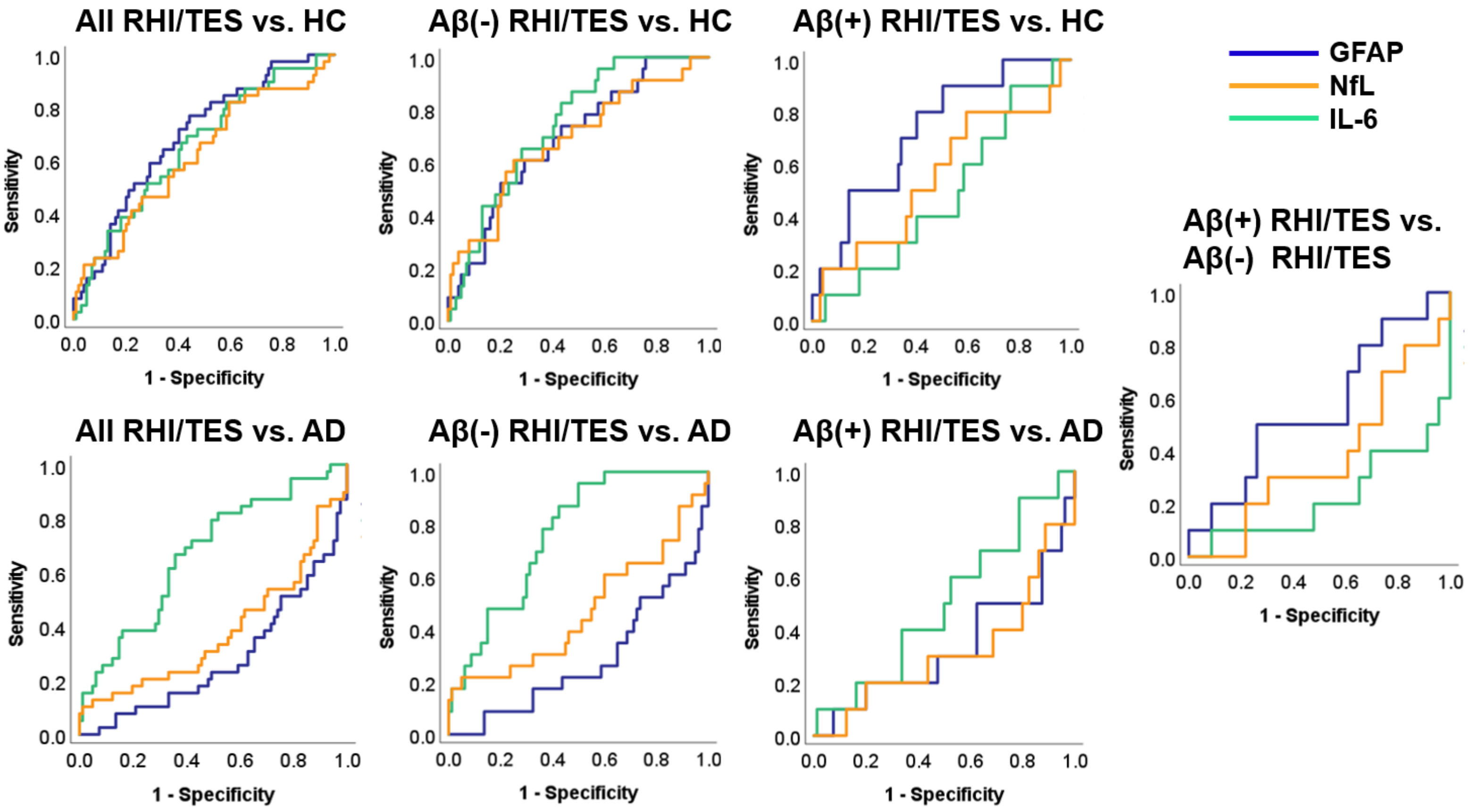

	GFAP		NfL		IL-6	
	HC	*AD*	*HC*	*AD*	*HC*	*AD*
**All RHI/TES**	0.69 (0.60-0.79)	0.28 (0.18-0.38)	0.62 (0.51-0.72)	0.38 (0.27-0.49)	0.65 (0.55-0.75)	0.67 (0.57-0.77)
**Aβ[-] RHI/TES**	0.69 (0.58-0.81)	0.28 (0.16-0.40)	0.68 (0.56-0.81)	0.47 (0.32-0.62)	0.74 (0.65-0.84)	0.76 (0.66-0.86)
**Aβ[+] RHI/TES**	0.73 (0.58-0.87)	0.33 (0.13-0.53)	0.55 (0.35-0.75)	0.32 (0.12-0.51)	0.48 (0.30-0.65)	0.50 (0.31-0.68)
**Aβ[+] vs. Aβ[-]**	0.57 (0.56)		0.37 (0.17-0.58)		0.22 (0.03-0.42)	

Supplemental Figure 7: Area under the curve (AUC) analysis showing differentiation of RHI/TES group(s) from healthy control (HC) and Alzheimer's disease (AD) cohorts. Age- and sex-adjusted plasma biomarker levels were calculated based on demographic effects observed in the HC group. For all comparison, the RHI/TES group is the positive state (i.e., AUC > 0.5 reflects higher concentrations associated with RHI/TES classification). For the within RHI/TES comparison, the Aβ(+) RHI/TES group is the positive state. The table shows the AUC values with 95% confidence intervals for all pairwise comparisons.
