# Supplementary material for: Alzheimer’s pathology is associated with altered cognition, brain volume, and plasma biomarker patterns in traumatic encephalopathy syndrome
Source: Alzheimers Res Ther. 2023 Jul 21;15:126. doi: 10.1186/s13195-023-01275-w (PMC10360257; doi:10.1186/s13195-023-01275-w)
Supplement: Supplementary file 2 — Additional file 2. [file 13195_2023_1275_MOESM2_ESM.pdf]

Supplemental Methods

Voxel-Wise Structural Neuroimaging

T1-weighted structural magnetic resonance imaging (MRI) scans were obtained on a 3.0 Tesla Siemens TIM Trio (35% of sample) or a 3.0 Tesla Siemens Prisma Fit (65% of sample) scanner. Magnetization prepared rapid gradient-echo (MPRAGE) sequences were used to obtained whole brain T1-weighted images (TR/TE/TI=2300/2.98/ 900 ms, α=9°; TR/TE/TI=2300/2.9/ 900 ms, α=9°). The field of view was 240x256mm, with 1x1 mm in-plane resolution and 1mm slice thickness and sagittal orientation for both sequences. Before processing, all T1-weighted images were visually inspected for quality control and those with excessive motion or image artifact were excluded. Magnetic field bias was corrected using the N3 algorithm [1].

Whole brain voxel-based morphometry (VBM) analysis was performed using Statistical Parametric Mapping (SPM12, Wellcome Centre for Human Neuroimaging, London, United Kingdom) software implemented within Matlab (MathWorks, R2023a). Pre-processing steps were completed using the SPM12 DARTEL (Diffeomorphic Anatomical Registration Through Exponentiated Lie Algebra) toolbox, which leverages a non-linear registration approach [2].

Images were bias-corrected and segmented into grey matter, white matter, and cerebral spinal fluid (CSF) tissue. Segmentations were visually inspected to ensure quality. Total Intracranial Volume (TIV), used to estimate head size, was created using the sum of grey matter, white matter, and CSF tissue volume [3]. An average grey matter mask was created using only healthy control sample subjects.

DARTEL was used to generate a sample-specific group template, then individual images were warped to that group template. Afterward, images in DARTEL space were normalized through linear registration to MNI (Montreal Neurological Institute) space and smoothed by 8 mm full-width-at-half-maximum Gaussian kernel filter.

PET Neuroimaging

Participants were scanned on one of two scanners: a Siemens Biograph 6 PET/CT scanner or ECAT PET scanner at the Lawrence Berkeley National Laboratory. Attenuation correction was performed using either an emission scan (ECAT) or low-dose CT scan (Biograph). PET acquisition and processing procedures are also detailed elsewhere[4].

Amyloid-PET was performed with either [11C]Pittsburgh Compound B (PIB, injected dose: ~15mCi) or [18F]Florbetapir (FBP; injected dose: ~10mCi). A dynamic PIB-PET scan was acquired for 90min immediately after intravenous PIB injection. FBP-PET acquisition included four 5-min frames between 50-70min post injection. PIB scans were reconstructed using an ordered subset expectation maximum algorithm with weighted attenuation and were smoothed using a 4 mm Gaussian kernel with scatter correction (calculated image resolution 6.5 × 6.5 × 7.25 mm using Hoffman phantom). FBP scan reconstruction followed ADNI protocols (http://adni.loni.usc.edu/methods/).

Reconstructed PET frames were realigned for motion correction and coregistered with reslicing to participants' T1-weighted MRIs. Mean count images were converted into voxel-wise Standardized Uptake Value Ratio (SUVR) images (FBP-PET and PIB-PET, 50-70min; FTP-PET, 80-100min) using tracer specific reference regions defined using Freesurfer-derived MRI parcellation and the SUIT atlas, as previously defined[5]. Tracer specific reference regions were whole cerebellum for FBP, cerebellar gray matter for PIB, and inferior cerebellar gray matter for FTP. PET images were warped to MNI space with the MRI-based transformations. Finally, PET images were differentially smoothed to obtain about the same final resolution (i.e., about 8 mm^3 isotropic).

FBP-PET and PIB-PET SUVR images were centrally read as positive or negative at UCSF based on visual assessment[6]. SUVRs were then converted to Centiloids (CLs) scale to

harmonize data across the two tracers[7-9]. A value of 100 CLs corresponds with the average degree of amyloid deposition observed in patients diagnosed with Alzheimer's disease dementia [10].

Tau-PET was performed using [18F]Flortaucipir (FTP; injected dose: ~10mCi; N=78). FTP-PET scan acquisition varied in their timing, although always including the 80–100min post injection window. FTP images were reconstructed using an ordered subset expectation maximum algorithm with weighted attenuation and were smoothed using a 4 mm Gaussian kernel with scatter correction (calculated image resolution 6.5 × 6.5 × 7.25 mm using Hoffman phantom). To quantify Flortaucipir binding, we extracted SUVR values from the whole cortex. An overall cortical SUVR was calculated with FTP-PET positivity defined as SUVR>1.27 [11].

Neuropathological Assessment

Two patients with TES had their beta-amyloid status determined via autopsy (1.1 and 7.7 year intervals between antemortem assessment and autopsy) using standardized sampling and staining protocols in the UCSF Neurodegenerative Disease Brain Brank as described elsewhere [12, 13]. Sampling procedures followed recommended guidelines for CTE, AD, FTLD, and synucleinopathies classification [14-16]. AD burden (Aβ plaques and AD tau tangles) was defined as "None," "Low," "Moderate," or "High" AD neuropathologic changes (ADNC) based on current NIA-AA criteria [16]. Both of these patients had frequent neuritic and diffuse plaques (CERAD A2/C3 and A3/C3; Braak stage not considered due to reliance on Aβ-PET only for patients characterized during life). One additional patient with TES underwent autopsy, which confirmed antemortem Aβ-PET positivity status (4.2 year interval; CERAD A3/C3). Relevant to the convenience sample of 12 patients with TES who were evaluated at autopsy, CTE severity was defined according to McKee staging criteria [17] and as "High" or "Low" based on recently

proposed classification methods that account for the number of brain regions with CTE-tau deposition (regardless of burden/density) [14]. Brains that were considered free of CTE pathology during initial autopsy evaluation were reexamined with additional regions reviewed for CTE tau pathology, per recent consensus group recommendations [14].

Supplemental References

1. Sled, J.G., A.P. Zijdenbos, and A.C. Evans, *A nonparametric method for automatic correction of intensity nonuniformity in MRI data.* IEEE transactions on medical imaging, 1998. **17**(1): p. 87-97.
2. Ashburner, J., *A fast diffeomorphic image registration algorithm.* Neuroimage, 2007. **38**(1): p. 95-113.
3. Malone, I.B., et al., *Accurate automatic estimation of total intracranial volume: a nuisance variable with less nuisance.* Neuroimage, 2015. **104**: p. 366-72.
4. Iaccarino, L., et al., *Spatial Relationships between Molecular Pathology and Neurodegeneration in the Alzheimer's Disease Continuum.* Cereb Cortex, 2021. **31**(1): p. 1-14.
5. Baker, S.L., A. Maass, and W.J. Jagust, *Considerations and code for partial volume correcting [18F]-AV-1451 tau PET data.* Data in brief, 2017. **15**: p. 648-657.
6. Lesman-Segev, O.H., et al., *Diagnostic Accuracy of Amyloid versus (18) F-Fluorodeoxyglucose Positron Emission Tomography in Autopsy-Confirmed Dementia.* Ann Neurol, 2021. **89**(2): p. 389-401.
7. Royse, S.K., et al., *Validation of amyloid PET positivity thresholds in centiloids: a multisite PET study approach.* Alzheimers Res Ther, 2021. **13**(1): p. 99.
8. Salvadó, G., et al., *Centiloid cut-off values for optimal agreement between PET and CSF core AD biomarkers.* Alzheimers Res Ther, 2019. **11**(1): p. 27.
9. La Joie, R., et al., *Multisite study of the relationships between antemortem [(11)C]PIB-PET Centiloid values and postmortem measures of Alzheimer's disease neuropathology.* Alzheimers Dement, 2019. **15**(2): p. 205-216.
10. Klunk, W.E., et al., *The Centiloid Project: standardizing quantitative amyloid plaque estimation by PET.* Alzheimers Dement, 2015. **11**(1): p. 1-15.e1-4.
11. Maass, A., et al., *Comparison of multiple tau-PET measures as biomarkers in aging and Alzheimer's disease.* Neuroimage, 2017. **157**: p. 448-463.
12. Kim, E.J., et al., *Mixed TDP-43 proteinopathy and tauopathy in frontotemporal lobar degeneration: nine case series.* J Neurol, 2018. **265**(12): p. 2960-2971.
13. Tartaglia, M.C., et al., *Sporadic corticobasal syndrome due to FTLD-TDP.* Acta Neuropathol, 2010. **119**(3): p. 365-74.
14. Bieniek, K.F., et al., *The Second NINDS/NIBIB Consensus Meeting to Define Neuropathological Criteria for the Diagnosis of Chronic Traumatic Encephalopathy.* J Neuropathol Exp Neurol, 2021.
15. Mackenzie, I.R., et al., *Nomenclature and nosology for neuropathologic subtypes of frontotemporal lobar degeneration: an update.* Acta Neuropathol, 2010. **119**(1): p. 1-4.

16. Hyman, B.T., et al., *National Institute on Aging-Alzheimer's Association guidelines for the neuropathologic assessment of Alzheimer's disease.* Alzheimers Dement, 2012. **8**(1): p. 1-13.
17. McKee, A.C., et al., *The spectrum of disease in chronic traumatic encephalopathy.* Brain, 2013. **136**(1): p. 43-64.
